# Supplementation with okra combined or not with exercise training is able to protect the heart of animals with metabolic syndrome

**DOI:** 10.1038/s41598-023-28072-7

**Published:** 2023-01-26

**Authors:** Moisés Felipe Pereira Gomes, Elizabeth de Orleans Carvalho de Moura, Naiara Magalhães Cardoso, Graziele Aparecida da Silva, Ana Carolina Cardoso dos Santos, Fernanda Samantha de Souza, Débora Estadella, Rafael Herling Lambertucci, João Henrique Ghilardi Lago, Alessandra Medeiros

**Affiliations:** 1 Department of Bioscience, Universidade Federal de São Paulo (UNIFESP), R. Silva Jardim, 136 - Vila Matias, Santos, SP 11015-020 Brazil; 2 Center for Applied Social Sciences and Health, Universidade Católica de Santos (Unisantos), Av. Conselheiro Nébias, 300, Vila Matias, Santos, SP 11015-002 Brazil; 3 Institute of Environmental, Chemical and Pharmaceutical Sciences, Universidade Federal de São Paulo (UNIFESP), Rua Prof. Artur Riedel, n° 275, Eldorado, Diadema, SP 09972-270 Brazil; 4 Center of Natural and Human Sciences, Universidade Federal Do ABC, Av. Dos Estados, 500, Bangú, Santo André, SP 09210-580 Brazil

**Keywords:** Cardiology, Metabolic diseases

## Abstract

The metabolic syndrome (MetS) is a clinical manifestation strongly associated with cardiovascular disease, the main cause of death worldwide. In view of this scenario, many therapeutic proposals have appeared in order to optimize the treatment of individuals with MetS, including the practice of exercise training (ET) and the consumption of okra (O). The aim of the present study was to evaluate the effect of O consumption and/or ET in animals with MetS. In all, 32 male Zucker rats (fa/fa) at 10 weeks old were randomly distributed into four groups of 8 animals each: MetS, MetS+O, MetS+ET and MetS+ET+O, and 8 lean Zucker rats (fa/ +) comprised the control group. Okra was administered by orogastric gavage 2x/day (morning and night, 100 mg/kg), 5 days/week, for 6 weeks. The ET was performed on a treadmill 1x/day (afternoon), 5 days/week, 60 min/day, in an intensity of 70% of maximal capacity, for the same days of O treatment. It was found that, O consumption alone was able to promote improved insulin sensitivity (MetS 93.93 ± 8.54 mg/dL vs. MetS+O 69.95 ± 18.7 mg/dL, *p* ≤ 0.05, d = 1.65, CI = 50.32 −89.58, triglyceride reduction (MetS 492.9 ± 97.8 mg/dL vs. MetS+O 334.9 ± 98.0 mg/dL, *p* ≤ 0.05, d = 1.61, CI = 193.2–398.7). In addition, it promoted a reduction in systolic blood pressure (MetS 149.0 ± 9.3 mmHg vs. MetS+O 132.0 ± 11.4 mmHg, *p* ≤ 0.05, d = 1.63, CI = 120–140), prevented an increase in cardiac collagen (MetS 12.60 ± 2.08% vs. MetS+O 7.52 ± 0.77%, *p* ≤ 0.05, d = 3.24, CI = 6.56–8.49). When associated with ET, the results were similar. Thus, we conclude that O consumption combined or not with aerobic ET can have a protective effect on the cardiac tissue of rats with MetS.

## Introduction

Health campaigns are held all over the world to raise awareness about obesity and the health problems associated with it, since it is responsible for promoting increased economic burdens and decreased life expectancy^1^, besides being one of the main components related to metabolic syndrome (MetS).

In fact, MetS is a disorder that manifests differently among individuals, but insulin resistance (IR) and/or central obesity are risk factors of greatest relevance to the development of MetS^2^. Besides being strongly associated with cardiovascular diseases (CVDs), MetS is related with increased mortality in young people and adults, as well as with the emergence of other diseases such as cancer, stroke, Alzheimer's disease and non-alcoholic steatohepatitis^3,4^.

In general, the medical expense of someone with MetS is 1.6 higher than that of individuals who did not develop the MetS. Moreover, for each component that constitutes the MetS there is an increase of about 26% in the value^5^. Therefore, lifestyle modification, through an adequate diet and regular exercise training (ET), is strongly suggested, in order to promote the maintenance of these individuals’ health^6^.

In this sense, aerobic ET has become an effective strategy, since several studies have shown that besides being a low cost, adjunct therapeutic approach^7^, it can improve metabolic aspects, promoting changes, such as: reduction of the glycemic index in diabetics, reduction of blood pressure and weight in obese people^8–10^.

In addition to ET being a low-cost approach, its effectiveness for disease prevention^11^ and treatment^12,13^ is well established in the literature. According to Ruberti et al., moderate-intensity aerobic ET is capable of promoting reduction of cardiometabolic alterations in cardiac tissue, reduction of collagen fraction after infarction and maintaining lower blood pressure levels compared to the sedentary group, although this difference is not statistically significant^14^.

Overall, few studies have evaluated the effects of ET on MetS *per se*^15,16^, most of which focus on the analysis of only one of the components of the syndrome. This is the case of STRRIDE, whose work has 302 participants, but the objective was limited to evaluating the impact of the amount of exercise on food consumption and body composition. In addition to the practice of ET, another habit that has been incorporated by people concerned with maintaining health or treating illnesses is the consumption of functional foods. According to Bailey, about 40% of the American adult population use some food for therapeutic purposes^17^. Thus, many foods have become targets for chemical and biological investigations to obtain new therapeutic ways, and these targets include okra.

Okra is the fruit of *Abelmoschus esculentus L. Moench* (Malvaceae), a plant native to Africa^18,19^, plays an important role in different Brazilian cuisines, a factor that encourages regional planting^20^. According to the nutritional table prepared by Pereira and Anção, in 100 g of raw okra the food will provide 33 kcal, 1.93 g of protein, 7.45 g of sugars, 3.2 g of dietary fiber, 299 mg of potassium, 0.026 g of unsaturated fiber and 0.027 g of polyunsaturated^21^.

Some studies show that okra or its components promotes an increase in reducing agents of reactive species^22^, has gastroprotective action^23^, modulates the stress response and has nootropic action^24^. In the study conducted by Fan et al., C54BL/6 mice were fed for 12 weeks with a high-fat diet, followed by another 2 weeks of follow-up, with the addition of some okra compounds commercially acquired (0.1% isoquercetin and quercetin 3-O) in the diet of the intervention group. The study demonstrated that the components were able to reduce body mass, total cholesterol (TC), LDL and blood glucose^25^.

Despite new drug arsenals for the treatment of MetS components, the economic factor may limit access to appropriate therapy. Therefore, our hypothesis is that okra could promote improvement in MetS components and consequently protect the heart of these animals from possible remodeling caused by MetS. Furthermore, okra could have a synergistic effect with exercise training.

This study aimed to verify if the administration of okra could improve the MetS components and consequently protect the heart of these animals. In addition, to assess whether there would be a synergistic effect with the ET protocol in Zucker rats, which is a model of MetS with severe obesity.

## Methods

### Animals

To obtain the sample size, the “resource equation” method described by Charan and Khantharia was used. This method consists of calculating to obtain the degree of freedom of the analysis of variance (E), using the formula “E = Total number of animals–Total number of groups”. Values of “E” smaller than 10 indicate the need to increase the number of animals per group ^26^. The “E” value of this study was equal to 35, that is, the appropriate sample size for carrying out the research.

Obese (fa/fa, n = 32) and lean fa/ + , n = 8 male Zucker rats were obtained from the Center of Experimental Models Development for Biology and Medicine of Universidade Federal de São Paulo (UNIFESP). All rats were 10 weeks old at the beginning of the experiment. Three animals were kept per cage with a standard diet for laboratory rats (Nuvilab CR1, for every 100 g of feed there were: carbohydrates 52.50 g, protein 22 g, lipids 4 g, 334 kcal/100 g) and water ad libitum. The ambient temperature was maintained between 22–23 °C and a 12:12 h light/dark cycle was adopted, with the light cycle starting at 8 p.m. All animals remained in the vivarium for one week, in order to acclimatize to the environment, reducing possible stress.

All experiments were carried out in accordance with the Guidelines and Brazilian Guide for the Creation and Use of Animals for Teaching and Research Activities and Conducted, after approval by the Research Ethics Committee of UNIFESP (CEUA 6912291116). The obese rats (fa/fa) were randomly separated in four groups of 8 animals each: Metabolic Syndrome (MetS), MetS+Okra (MetS+O), MetS+Exercise Training (MetS+ET) and MetS+Exercise Training+Okra (MetS+ET+O). Lean rats (fa/ +) comprised the Control group (CTL). All animals had their tails marked with permanent colored brushes and were placed in boxes with animals that received the same intervention in order to facilitate the identification of animals correctly and reduce the confusion rate.

During the research there was no adverse event with the animals. All animals were housed together, in an environment enriched with plastic cylinders and paper towels, to avoid stress due to social isolation.

### Training protocol

During 4 days, the animals performed exercise sessions on a treadmill with speed between 5 and 15 m/min, lasting 20 min in order to adapt to the treadmill. On the fifth day, a physical exercise tolerance test was performed as described by Moraes-Silva^27^, which consisted of a staggered protocol with an initial speed of 3 m/min for 5 min for warm-up and increments in the treadmill speed of 3 m/min every 3 min, until the animal is exhausted. The aerobic ET protocol was held for 6 weeks (in the same days of okra administration), 1x/day (afternoon), 5 days/week, 60 min/day, with an intensity of 70% of the speed obtained in the maximum effort test. Halfway through the protocol (3 weeks), a new effort test was performed only in the groups submitted to ET in order to adapt the intensity of the ET. However, to assess the effect of interventions on tolerance to exertion, all of the animals were subjected to this test at the beginning and at the end of the intervention period.

### Okra administration

Okra (*Abelmoschus esculentus L. Moench*) samples were sprayed and lyophilized to be administered by orogastric gavage. The animals received a concentrations of 100 mg/kg diluted in 1 ml of filtered water, 2×/day (morning and night, total 200 mg/kg animal/day), 5 days/week, for the 6 weeks of the experimental protocol (at the same period the exercise training protocol). The groups that did not receive okra were gavaged with 1 ml of filtered water so that all animals were subjected to the same conditions.

### Murine measurements and blood pressure assessment

The body mass of all groups was assessed using semi-analytical scales (Gehaka). Blood pressure (BP) and heart rate (HR) were evaluated by plethysmography of the tail and by a specific system for rats (Visitech Systems: BP-2000–Series II–Blood Pressure Analysis System), which has a close correlation with direct intra-arterial measurements (*p* < 0.02, *r* = 0.98)^28^. These assessments were carried out in the last week of the experimental protocol, on day when the rats were not subjected to training sessions.

### Assessment of serum markers of metabolic syndrome

In the last week of the interventions period, blood was collected from caudal vein to evaluate triacylglycerol (TG), total cholesterol (TC) and glycemia after 12-h fasting. The analyses were performed with specific reagent strips for the Accutrend ® Plus device.

Each test strip has a test area that contains detection reagents. When blood is applied, a chemical reaction occurs and the test area changes color. The device registers this color change and converts the measurement signal into the result displayed using the data previously entered through the code strip.

### Insulin tolerance test and glucose tolerance test

At the end of the 6th week of the intervention protocol, insulin sensitivity was assessed using the Insulin Tolerance Test (ipITT) and the Glucose Tolerance Test (ipGTT). In the analysis of the ipITT, the animals remained fasting for 6 h before blood was collected through a cut in the tail. The blood glucose was evaluated with the use of a glucometer (time zero) and after the administration of insulin (2 IU/kg, ip) blood glucose was analyzed again at 5, 10, 15 and 30 min. After 72 h of ipITT, the ipGTT test was performed. The animals remained fasting for 8 h and the same procedure was performed for blood collection. However, after time zero, glucose (2.0 g/kg of body mass) was injected intraperitoneally and blood samples were collected at times 30, 60, 90 and 120 glycemia^29^.

### Euthanasia

The animals were anesthetized with thiopental (30 mg/kg, ip) and lidocaine (5 mg/kg, ip) and euthanized by beheading in guillotine, a fast method and free of prolonged suffering. The animals' blood was collected in dry tubes and centrifuged (3000 rpm for 15 min at 8 °C). At the end of the centrifugation, the plasma was separated into 1.5 ml conical tubes and stored in a biofreezer at −20 °C for further analysis. The visceral, retroperitoneal and epididymal adipose tissue deposits as well as liver and heart ventricles were dissected out and weighed, and the weight of the latter was corrected by the animal's tibia length.

### Alanine aminotransferase evaluation

The evaluation of alanine aminotransferase (ALT) was carried out with the ALT kit from LabTest, adapted to be performed in a 96-well microplate. Briefly, 200 µl of reagent 1 was pipetted into the wells + 25 µl of sample or calibrator from LabTest, and the plate was placed in an oven at 37 °C for 5 min. Then, 50 µl of reagent 2 was pipetted into each well and the absorbance reading was taken immediately at 340 nm (Epoch, Life Technologies), and after 2 min the second reading was taken. The zero was performed with distilled water.

### Aspartate aminotransferase evaluation

The evaluation of aspartate aminotransferase (AST) was performed using the AST kit from LabTest, adapted to be performed in a 96-well microplate. Briefly, 200 µl of reagent 1 was pipetted into the wells + 12.5 µl of sample or calibrator from LabTest, and the plate was placed in an oven at 37 °C for 5 min. Then, 50 µl of reagent 2 was pipetted into each well and the absorbance reading was taken immediately at 340 nm (Epoch, Life Technologies), and after 2 min the second reading was taken. The zero was performed with distilled water.

### Cardiac structure analysis

The left ventricles (LV) of the animals were transversally dissected and fixed in 4% buffered formalin solution for 24 h, with subsequent washing in saline solution (0.9% NaCl) and stored in 70% alcohol for a week. Tissue dehydration was performed in a 1-h immersion in solutions with increasing alcohol concentration (80, 90, and 100%). Subsequently, tissue clarification was performed in Xylol, two immersions lasting 1 h each, and finally the inclusion in paraffin. 5 µm sections were made and they were stained with hematoxylin–eosin. Only nucleated cardiomyocytes from the area of the longitudinal section were included in the analysis of cardiac fiber diameter. The quantification of ventricular fibrosis was performed using picrosirius staining, which allows the analysis of collagen fibers arranged parallel to the cardiac fibers. These measurements were analyzed with a computer- assisted morphometric system (Leica Quantimet 500, Cambridge, UK, England).

### Interleukins of hepatic and cardiac tissue

Interleukin-6 (IL-6), interleukin-10 (IL-10) and tumor necrosis factor-α (TNF-α) cytokines were analyzed using R&D System kits. For this, 100 mg of liver tissue and 50 mg of heart tissue were homogenized in 1 ml of protein extraction solution (5% BSA in sterile PBS, pH 7.2, 0.017% phenylmethylsulfonyl fluoride, 0.048% chloride benzethonium, 0.37% EDTA, 0.002% aprotinin)^30,31^. The homogenate was centrifuged at 10,000 rpm for 10 min at 4 °C. The infranatant was separated and used for analysis following the manufacturer's recommendations.

### Total antioxidant state of hepatic and cardiac tissue

Hepatic and myocardial total antioxidant statuses (TAS) were measured using the method described by Erel, but with some modifications^32^. For this, some solutions were prepared: solution 1, of sulfuric acid, pH 5.8 (940 ml of acetate sodium buffer solution at 0.4 mol/L, pH 5.8, plus 60 ml of glacial acetic acid solution 0.4 mol/L); solution 2 with a pH of 3.6 (75 ml of 30 mmol/L sodium acetate solution, pH 3.6, plus 925 ml of 30 mmol/L glacial acetic acid); solution 3 was prepared by mixing 37.5 ml of solution 1 to 462.5 ml of solution 2; solution 4 was prepared by diluting hydrogen peroxide 35% in solution 3 (2 mmol/L). Then, solution 5 was prepared by adding ABTS (10 mmol/L, pH 3.7) to solution 4. The test was carried out with 200 µL of solution 1 + 05 µL of sample + 20 µL of solution 5. Absorbance was read after 5 min, at the 620 nm wavelength.

### Total oxidant state of hepatic and cardiac tissue

Hepatic and myocardial total oxidant statuses (TOS) were measured using the method described by Erel^33^. For this, some solutions were prepared. Solution 1, of sulfuric acid, pH 1.75, with 150 uM xynenol Orange, 140 mM NaCl, and 1.35 M. Solution 2 consists of 10 mM of o o-Dianisidine dihydrochloride, 5 mM ferrous sulfate ammonia, 25 mM sulfuric acid. The test is performed with 225 μL of solution 1 + 35 μL of sample + 11 μL of solution 2. Absorbance was read after 5 min, at 560 nm in the first reading and at 800 nm after another 5 min.

### Thiobarbituric acid reactive substances (TBARS) analysis in hepatic and cardiac tissue

Sodium phosphate solution (0.1 M) was used as an extraction buffer and the analysis was performed according to Winterbourn^34^. In all, 50 mg of the left ventricle (LV) and 100 mg of liver were homogenized in saline buffer and the protein precipitate was removed by centrifugation (12,000 × G for 10 min). 500 µL of supernatant was mixed with 500 µL thiobarbituric acid (1% in 50 mM NaOH) and 500 ul HCl at 25%. The samples were heated for 10 min, followed by refrigeration. TBARS was extracted with 1.5 ml of butanol and centrifuged (12,000 × G for 10 min) and absorbance was measured at 532 nm.

### Hepatic glycogen analysis

To analyze the liver's glycogen content, the method described by Moura^35^ was used. The tissue was digested in a potassium hydroxide solution (30%) at 100 °C, and glycogen was precipitated after the addition of ethanol (70%) and centrifugation at 3500 rpm for 30 min. The supernatant was discarded, and the precipitate was washed with trichloroacetic acid (5%). Glycogen concentration was estimated with a solution of anthrone (0.2%) and sulfuric acid (95%), after reading the absorbance with at 490 nm.

### Statistical analysis

The data collected during the study were tabulated and given another name, so that one of the authors could perform the analysis blindly. The software GraphPad Prism 7.0 was used for the statistical analysis. We adopted the Kolmogorov–Smirnov test to verify normality and ANOVA (one or two-way) and *post-hoc* Bonferroni. For effect size, Cohen's “d” was reported using Becker's Effect Size Calculators^36^. The results appear as mean ± standard deviation (SD) and *p* ≤ 0.05 was adopted as the limit of statistical significance and confidence interval (CI) of 95%.

## Results

### Effect of interventions on physical effort tolerance

**Figure 1:**
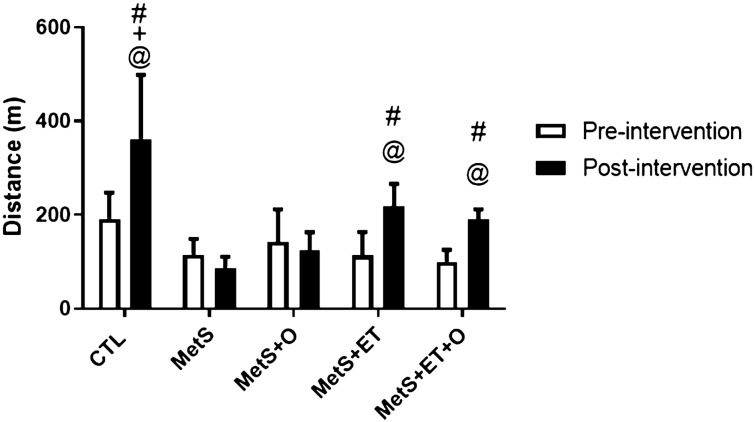
Exercise capacity represented by maximal distance run. CTL–Control, MetS–Metobolics Syndrome, MetS+O–Metabolic Syndrome and Okra, MetS+ET–Metabolic Syndrome and Exercise Training, MetS+ET+O–Metabolic Syndrome and Exercise Training and Okra. @ In the same group, #Diffrent from MetS post-intervention, +Different from MetS+O post-intervation. *p* ≤ 0.05.

Regarding the data obtained in the effort tolerance test (Fig. 1), there were no differences between the groups in the pre-intervention period. As expected, all groups that underwent ET showed improved performance in the post-intervention as compared to the pre-intervention moment. The control group also showed improved performance. In addition, the same groups performed better after training as compared to the MetS groups.

### Effect of interventions on MetS parameters

**Figure 2:**
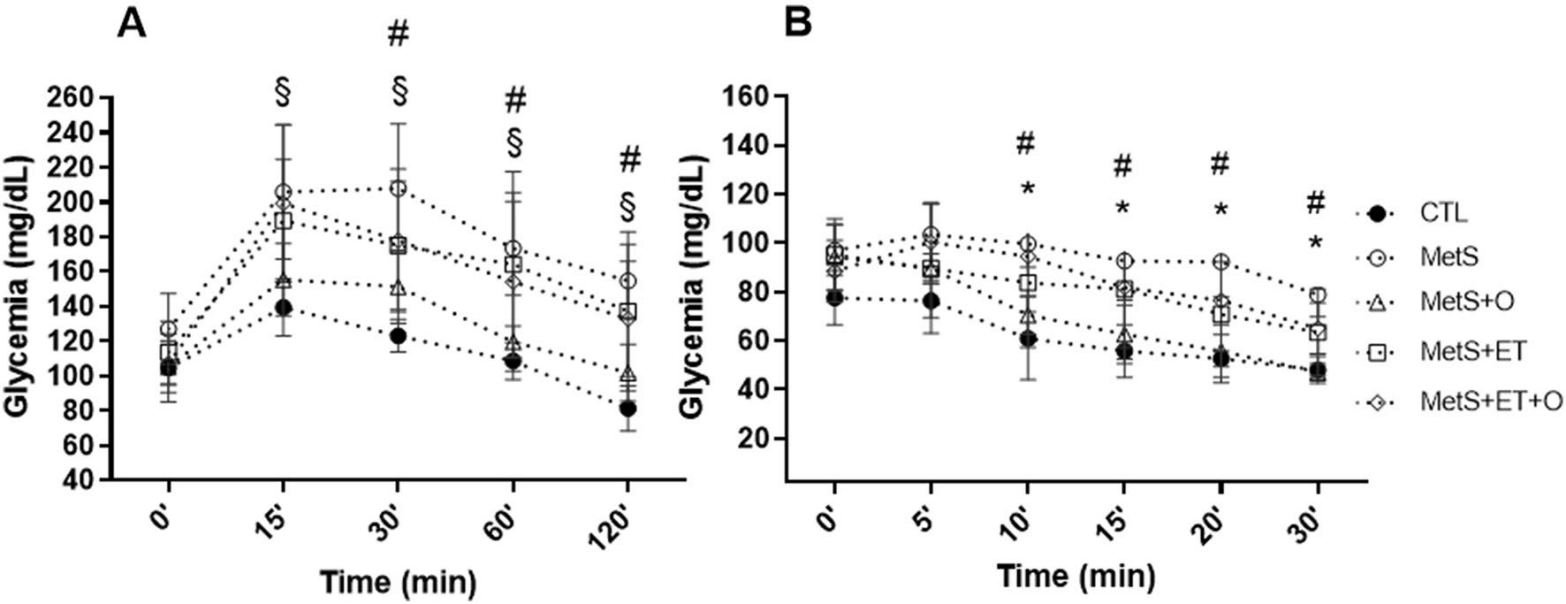
Analysis of glucose and insulin tolarance. (**a**) Glycemia during ipGTT test. (**b**) Glycemia during ipITT test. CTL–Control, MetS– Metabolic Syndrome. MetS+O–Metabolic Syndrome and Okra, MetS+ET–Metabolic Syndrome and Exercise Training, MetS+ET+O–Metabolic Syndrome and Exercise Training and Okra. §MetS+ET and MetS+ET+O different from CTL, *MetS different from CTL, # MetS+O different MetS. *p* ≤ 0.05.

As for the effects of interventions on MetS parameters, we found that the MetS, MetS+ET and MetS+ET+O groups showed greater glucose intolerance compared to the CTL group (Fig. 2a). However, the group that received only okra showed significantly lower values than the MetS group from 30 min (Fig. 2a). Regarding the insulin tolerance test, we found that only the MetS group had worse insulin sensitivity than the CTL (Fig. 2b). Only the Mets+O group showed better sensitivity to insulin than the MetS group. Although the groups that performed ET had lower values than the MetS group, there was no significant difference, but it is important to note that there was also no significant difference in relation to CTL.

We observed that all groups composed of animals with MetS had greater body mass and greater amount of visceral fats compared to the CTL group (Table 1). Although HR is not a parameter to diagnose MetS, we found that only the MetS group had a significant increase compared to CTL group. The groups that received okra and/or performed ET had HR similar to the CTL group. However, only the groups that received okra supplementation had significantly lower values than the MetS group (Table 1).

**Table 1:** MetS parameters and Heart Rate. CTL–Control, MetS–Metabolic Syndrome, MetS+O–Metabolic Syndrome and Okra, MetS+ET–Metabolic Syndrome and Exercise Training, MeTS+ET+O–Metabolic Syndrome and Exercise Training and Okra. SBP–Systolic Blood Pressure, DBP–Diastolic Blood Pressure. *Different from CTL, # Different from MetS, + Different from MetS+O. *p* ≤ 0.05.

	CTL	MetS	MetS+O	MetS+ET	MetS+ET+O
Body weight (g)	330.6 (± 29.3)	504.5(± 82.6)*	515.8 (± 51.4)*	531.7 (± 47.2)*	515.0 (± 54.6)*
Sum visceral fat (g/100 g)	2.30 (± 0.5)	7.31 (± 1.2)*	7.23 (± 0.8) *	7.34 (± 1.3)*	8.04 (± 0.5)*
Heart rate (bpm)	379.9 (± 13.1)	416.7 (± 19.3)*	375.0 (± 26.1)^#^	387.6 (± 9.5)	372.0 (± 19.8)^#^
Blood pressure (mmHg)					
SBP	131.3 (± 2.9)	149.0 (± 9.3)*	132.0 (± 11.4)^#^	129.2 (± 14.9)^#^	135.2 (± 5.4)^#^
DBP	73.44 (± 8.3)	89.57 (± 5.8)*	81.0 (± 9.9)	90.14 (± 10.7)*	81.88 (± 10.5)
Analysis of serum (mg/dL)					
Glycemia fasting	94.30 (± 9.0)	111.4 (± 13.6)	102.3 (± 16.7)	112.0 (± 14.1)	107.9 (± 10.7)
Triacylglycerol	92.45 (± 18.8)	492.9(± 97.8)*	334.9 (± 98.0)*^#^	465.0 (± 113.3)*^+^	412.0 (± 54.4)*
Total cholesterol	157.1 (± 7.6)	162.4 (± 9.7)	159.8 (± 7.4)	160.8 (± 5.3)	162.9 (± 11.8)

In relation to BP (Table 1), the animals that comprised the MetS group presented increased values of both systolic (SBP) and diastolic blood pressure (DBP). All interventions protocols (ET, supplementation with okra or both interventions together) contributed to promote significantly lower values of SBP in relation to the MetS group. On the other hand, DBP levels remained high in the MetS+ET groups compared to CTL and only the groups that received okra (MetS+O and MetS+ET+O) had DBP levels similar to CTL.

Although there were no differences in fasting glucose and TC of the animals (Table 1), TG of all groups with fa/fa animals had a higher mean than the CTL group. On the other hand, MetS+O showed lower values than MetS, which did not happen in the groups with ET intervention. In addition, MetS+O presented smaller results than MetS+ET (Table 1).

### Effect of interventions on cardiac tissue

Regarding the effects of interventions on cardiac tissues, we observed that total masses and relative to tibia length of the left and right ventricles of groups composed of fa/fa animals presented higher values as compared to CTL (Table 2). The interventions did not have effect on those variables.

**Table 2:** Weight of the heart chambers CTL–Control. MetS–Metabolic Syndrome. MetS+O–Metabolic Syndrome and Okra. MetS+ET–Metabolic Syndrome and Exercise Training. MeTS+ET+O–Metabolic Syndrome and Exercise Training and Okra. * Different of CTL. *p* ≤ 0.05.

	CTL	MetS	MetS+O	MetS+ET	MetS + ET + O
LV gross weight (g)	0.42 (± 0.2)	0.75 (± 0.1)*	0.82 (± 0.0)*	0.79 (± 0.2)*	0.83 (± 0.0)*
LV weight/tibia (g/mm)	0.01 (± 0.0)	0.02 (± 0.0)*	0.02 (± 0.0)*	0.02 (± 0.0)*	0.02 (± 0.0)*
RV gross weight (g)	0.10 (± 0.0)	0.21 (± 0.0)*	0.21 (± 0.0)*	0.26 (± 0.0)*	0.02 (± 0.0)*
RV weight/tibia (g/mm)	0.001 (± 0.0)	0.006 (± 0.0)*	0.005 (± 0.0)*	0.007 (± 0.0)*	0.007 (± 0.0)*

**Figure 3:**
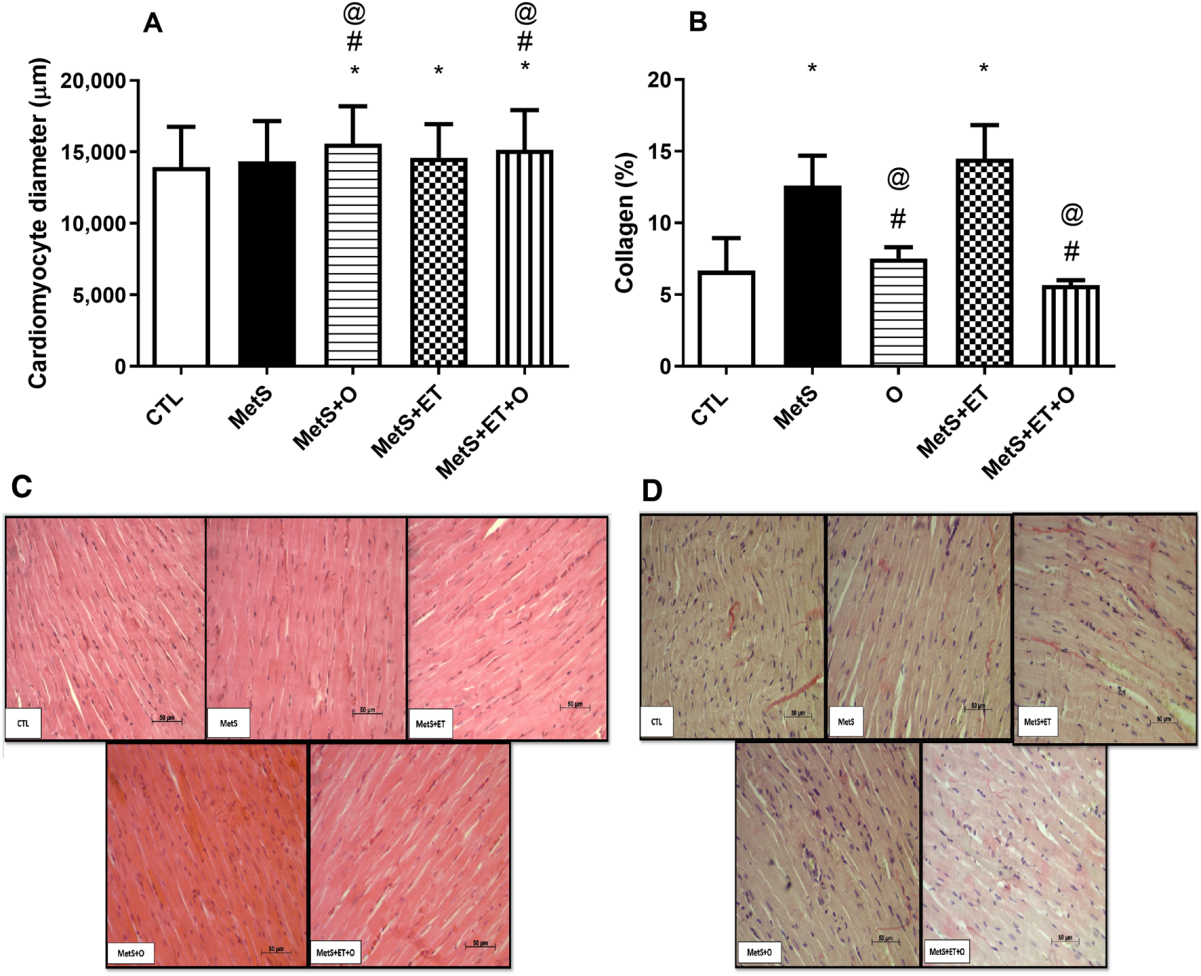
Histological analysis of the ventricles using hematoxylin and cosin staining technique of the groups. (**a**) Cardiomyocyte diameter: (**b**) Fraction of collagen in cardiac tissue. (**c**) Representative images of sections stained with hematoxylin and cosin. (**d**) Representative images of picrosirus-stained section. CTL–Control, MetS–Metabolic Syndrome, MetS+O–Metabolic Syndrome and Okra, MetS+ET– Metabolic Syndrome and Exercise Training, MetS+ET+O–Metabolic Syndrome and Exercise Training and Okra. *Different from CTL, #Different from MetS, @Differenct from MetS+ET, *p* ≤ 0.05.

Figure 3 shows the data obtained through the histological analysis of the LV. The diameter of cardiomyocytes of the MetS group was similar to CTL group. However, MetS+O (15,570 ± 2626 µm, d = 0.6, IC = 15,340–15,800), MetS+ET (14,584 ± 2355 µm, d = 0.25, IC = 14,377–14,791) and MetS+ET+O (15,146 ± 2786 µm, d = 0.43, IC = 14,901–15,390) were significantly larger compared to CTL (13,935 ± 2821 µm). In addition, it was found that okra groups had a larger diameter as compared to MetS (14,341 ± 2824 µm, IC = 14,094–4587) and MetS+ET (Fig. 3a).

Regarding the cardiac collagen fraction (Fig. 3b), MetS (12.60 ± 2.08%, d = 2.73, IC = 9.27–15.93) presented higher values than CTL (6.68 ± 2.25%, IC = 3.88–9.48). Only the groups that received okra supplementation (MetS vs. MetS + O 7.52 ± 0.77%, d = 3.24, IC = 6.56–8.49 and MetS vs. MetS+ET+O 5.66 ± 0.34%, d = 4.65, IC = 5.12–6.21) had a lower collagen content compared to MetS. The group that performed only the ET (MetS+ET 14.48 ± 2.35%) showed no change in this variable when compared to MetS group.

In the evaluation of cytokines in cardiac tissue, it can be seen in Table 3 that MetS had a lower concentration of IL-10 as compared to CTL and that none of the interventions was able to change this concentration. Regarding IL-6 concentration, although MetS has a higher concentration than CTL, this difference was not statistically significant. Only MetS+O had a significantly lower concentration of IL-6 compared to MetS. As for TNF-α, no significant differences were observed between groups (Table 3).

**Table 3:** Interleukin and analysis of oxidative stress in cardiac tissue. CTL–Control, MetS–Metabolic Syndrome, MetS+O–Metabolic Syndrome and Okra, MetS+ET–Metabolic Syndrome and Exercise Training, MeTS + ET + O–Metabolic Syndrome and Exercise Training and Okra. IL–10–Interleukin −10, IL–6–Interleukin–6, TNF–α–Tumor Necrosis Factor Alpha, MDA–Malondialdehyde, TAS–Total Antioxidant Status, TOS–Total Oxidant Status, OSI–Oxidative Stress Index. *Different from CTL, #Different from MetS. *p* ≤ 0.05.

	CTL	MetS	MetS+O	MetS+ET	MetS+ET+O
IL–10 (pg/µg)	191.1 (± 44.6)	52.2 (± 35.8)*	55.7 (± 17.9)*	34.6 (± 8.0)*	32.9 (± 28.6)*
IL–6 (pg/µg)	67.9 (± 8.0)	84.7 (± 27.5)	28.4 (± 8.0)^#^	37.3 (± 22.6)	45.5 (± 24.8)
TNF–α (pg/µg)	41.0 (± 13.6)	40.5 (± 10.1)	42.8 (± 7.2)	35.8 (± 12.0)	49.3 (± 12.1)
MDA nm (UI)	1.2 (± 0.1)	1.2 (± 0.1)	1.3 (± 0.2)	1.42 (± 0.2)	1.4 (± 0.2)
TAS (mmol Trolox equ./L)	1.4 (± 0.0)	1.6 (± 0.0)	1.4 (± 0.2)	1.7 (± 0.1)	1.5 (± 0.1)
TOS (mmol H_2_O_2_ equ./L)	10.9 (± 0.0)	11.0 (± 0.2)	10.9 (± 0.1)	10.9 (± 0.0)	10.7 (± 0.3)
OSI (TOS/TAS)	7.3 (± 0.3)	6.82 (± 0.4)	6.9 (± 0.6)	6.4 (± 0.3)	7.0 (± 0.7)

There were also no significant differences between groups in the oxidative stress variables assessed in cardiac tissue (Table 3).

### Effect of interventions on liver tissue

Although the groups composed of fa/fa animals had a greater mass of liver tissue than CTL animals, there was no difference in ALT and AST levels, indicating that there was no difference in liver function between groups (Table 3).

In relation to IL-10 analysis performed in the liver, there was no difference between MetS and CTL groups. Only MetS+O showed a higher IL-10 concentration than CTL (Table 3). IL-6 concentration was significantly higher in MetS than in CTL. Only the group treated in combination with okra supplementation plus ET (MetS+ET+O) had a lower IL-6 concentration compared to MetS, MetS+ET and MetS+O (Table 3). In oxidative stress variables investigated in the liver, there were no differences between MetS and CTL groups. Only MetS+ET+O had lower TAS values than CTL, MetS and MetS+ET, while TOS values were lower than in CTL and MetS. And although there was a reduction in the total oxidative rate in MetS+ET+O, the difference was not significant.

**Figure 4:**
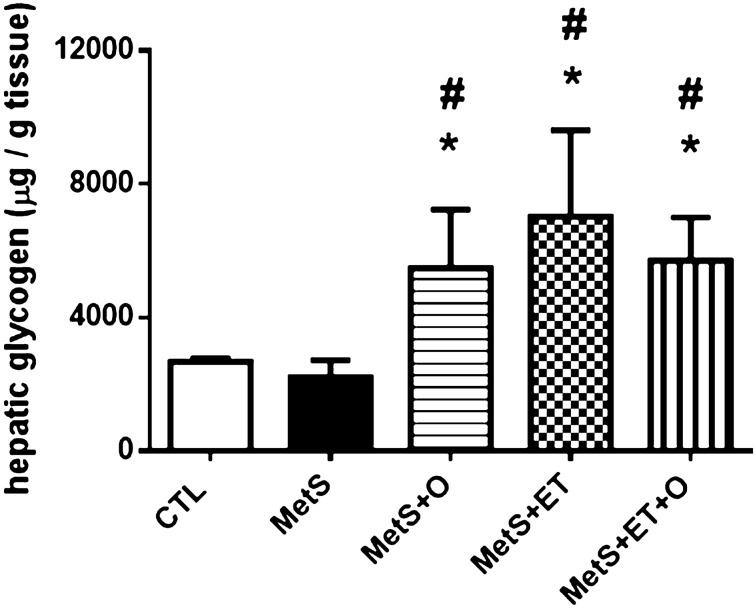
Analysis of hepatic glycogen. CTL–Control, MetS–Metabolic Syndrome, MetS+O–Metabolic Syndrome and Okra, MetS+ET–Metabolic Syndrome and Exercise Training, MetS+ET+O–Metabolic Syndrome and Exercise Training and Okra. *Different from CTL, #Diiferent from MetS. *p* ≤ 0.05.

Finally, Fig. 4 shows that the liver glycogen content was similar in MetS (2213 ± 514.3 µg/g, IC = 1395–3031) and CTL (2675 ± 103.0 µg/g, IC = 2567–2783), but the groups with some type of intervention showed higher concentration of this variable as compared to MetS (MetS vs. MetS+O 5.488 ± 1.746 µg/g, d = 9, IC = 3.320–7.657), (MetS vs. MetS+ET 7.024 ± 2.594 µg/g, d = 13.22, IC = 3.803–10.244) and (MetS vs. MetS+ET+O 5.708 ± 1.299 µg/g, d = 9.61, IC = 4.095–7.320).

## Discussion

Although some studies show satisfactory effects by reducing the risk factors of CVDs, the present study was the first demonstrating the direct effect of okra consumption on the heart, preventing accumulation of interstitial collagen of cardiomyocytes (Fig. 3) in MetS+O and MetS+ET+O. Moreover, the okra-supplemented groups showed larger diameter of cardiomyocytes (Fig. 3), explaining that although there is no difference in gross and relative weight of the cardiac chambers (Table 2), the composition of the chambers differs across the groups.

Prathapan et al. demonstrated that the ethanolic extract of *Boerhavia difusa* is rich in flavonoids, capable of reducing collagen deposition and mitigating cardiac fibrosis via inhibition of growth transforming factor β1 (TGF-β1)^37^. Similarly, Pan et al. showed that some flavones, such as scutellarin, are capable of modulating collagen production of cardiac fibroblasts in vitro^38^. The cardiac remodeling promoted by the increase in interstitial collagen is capable of generating cardiac dysfunctions, since the cardiac fiber's capacity to distend is reduced, causing impairment of the final diastolic volume^39^. In this sense, although interventions with ET and okra consumption did not promote the prevention of weight gain (Table 1), an independent risk factor for cardiac remodeling^37^, we found that MetS+O and MetS+ET+O obtained benefits, since okra prevented cardiac remodeling, which is an important predictor of mortality in humans^39–41^.

It is very important to highlight that the absence of a positive response to the interventions in this study, in the variables related to obesity, may be related to the fa/fa animal model, which has a genetic alteration that promotes leptin resistance^42,43^. In fact, another study performed with a fa/fa model showed that the combination of consumption of hydrolyzed lentil protein and high-intensity aerobic exercise on a treadmill and strength training did not contribute to the reduction of caloric consumption and body mass of the Zucker rats^44^.

The main mechanisms involved in this result may be related to the control of two other important risk factors for cardiac remodeling, which are systemic arterial hypertension (SAH) and IR. In Table 1, we see that all intervention groups (MetS+O, MetS+ET and MetS+ET+O) showed lower BP values than MetS. SAH promotes cardiac hypertrophy due to the hemodynamic overload imposed by the increase in afterload, causing sarcomere synthesis in parallel^45^. In addition, the upregulation of the renin–angiotensin–aldosterone system promotes activation of the angiotensin II receptor, which promotes increased cardiac fibrosis . Importantly, studies in the area of primary prevention of SAH show that the difference of 2 mmHg reduces the risk of mortality from stroke and cardiac events in up to 6% and 4%, respectively^46^.

The second mechanism associated with decreased cardiac collagen content in our work may be related to improved insulin sensitivity (Fig. 2b). In fact, type 2 diabetes mellitus (T2DM) is an independent risk factor for cardiac remodeling^39^. In addition, many studies have already shown that IR or T2DM are able to promote changes in cardiac tissue, namely: mitochondrial dysfunction, decreased sensitivity to Ca^2+^, oxidative stress, apoptosis, necrosis and increased collagen in the interstitial space^47–49^.

Generally, the cardiac remodeling arises in diabetic rats after 3 months of induction of the disease ^8^. It is well known that Advanced Glycation End (AGE) products synthesized during obesity and IR cooperate with cardiac fibrosis by interaction with the receptor of AGE (RAGE), which stimulates the release of pro-fibrotic growth factors, as well as collagen deposition^50^. In addition, AGEs promote stiffening of cardiac collagen through cross-links between type 1 collagen molecules^47^.

Besides the morphological changes in the heart, we found that cardiac excitability decreases in the intervention groups (HR in Table 1). Previous research has shown that body composition can influence the sympathovagal balance^51^, causing greater sympathetic activity according to the individuals' body mass index (BMI)^52^. Although the animals did not show a reduction in adiposity, there was an improvement of IR. We therefore believe that the reduction of metabolic stress caused by the improvement of glucose intolerance and IR (Fig. 2a and b) contributed to the HR decrease in the groups that received okra (MetS+O and MetS+ET+O). Sridhar had already described that diabetes can promote severe autonomic dysfunction, especially in the heart^53^. In addition, De Angelis explains that IR and increased blood glucose promote a hyperdynamic state of the sympathetic nervous system, which explains the increase in HR^54^. However, Young and Benton described that dietary interventions, such as: consumption of Mediterranean diet, omega-3 fatty acids, B-vitamins, probiotics and polyphenols are able to increase heart rate variability^55^.

The okra consumption appears to act synergistically to reduce sympathetic activity. According to Monal et al., rats that consumed 150 mg/kg of ethanolic okra extract showed a reduction in the RR interval on the electrocardiogram^56^, demonstrating lower sympathetic tone.

Vagal modulation is an important physiological mechanism in glucose homeostasis, and the most diabetic individuals develop some degree of autonomic dysfunction when developing the disease ^57^. According to Joseph et al., rats that underwent vagal stimulation showed a reduction in glucose levels and an increase in insulin secretion ^58^. Therefore, it is possible to infer that the consumption of okra may have helped in the vagal modulation of rats with MetS.

In the groups that underwent ET, studies show that aerobic exercise^59^ or resistance exercise^9^ are able to recover baroreflex activity and decrease sympathetic activity^60,61^, which would explain the reduction in BP levels and HR^62,63^.

There is still no consensus in the literature on which mechanisms are involved in the reduction of the sympathetic hyperdynamic state by ET. Among the possible hypotheses, it is conjectured that ET is capable of promoting neuroplasticity in neurons related to the control of the sympathetic nervous system^64,65^. Kramer et al., observed that spontaneously hypertensive rats submitted ET on a treadmill, had restoration of GABAergic activity in the caudal hypothalamus, and with it, the reduction of excitatory neuron firing^66^.

Although there was no significant improvement in inflammatory markers and oxidative stress in cardiac tissue (Table 3), except for the reduction of IL-6 in MetS+O (Table 3), it is important to highlight that for the first time IL-10 was evaluated in cardiac tissue from Zucker rats, and was observed an increase in concentration in all groups composed of fa/fa animals. In addition to IL-10 attenuating the induced oxide synthesis and apoptosis in cardiac tissue, studies have shown that IL-10 concentration in human serum is diminished in individuals with cardiac comorbidities^67,68^.

On the other hand, although liver tissue has presented increased IL-6 in most groups, we found that only the group with combined interventions (MetS+ET+O) showed a significant reduction in IL-6 and TOS, demonstrating a greater physiological reserve of liver tissue in the face of pathological stimuli. Moreover, we found that despite an environment more conducive to the inflammatory state, characterized by increased IL-6 levels, we observed that there was no change in liver glycogen content, which points to the hepatic protective effect that all of those who consumed okra and performed ET obtained during the protocol period^69^. On the other hand, the increase in the gross weight of the liver can be justified by the huge caloric consumption that this Zucker model has, since the use of high-calorie diets in animals can generate an increase in the gross weight of the liver^70^.

Finally, we found that the consumption of okra did not influence the final outcome of the animals in performing more physical effort (Fig. 1), different from the result reported by Xia, where the rats took longer to reach the state of fatigue during the maximum effort test after consumption of okra seeds^47^. On the other hand, Xia adjusted the doses of okra components, by standardizing the antioxidant activity found in each segment, which does not reflect the reality of okra consumption during meals. However, as expected, the groups that performed ET showed a significant improvement in the tolerance to exertion, confirming the effectiveness of the protocol used.

## Conclusion

Unlike other studies that used only the administration of chemically extracted phytochemicals^47,71^ or commercially available synthetic compounds^25^, in the present study we used lyophilized okra and we found that its consumption alone promoted a reduction in IR and TG, as reported in other studies. In addition, the effect of okra consumption on reducing BP, on increasing cardiomyocyte diameter and on preventing collagen deposition in the cardiac tissue of male rats with MetS was demonstrated for the first time. When ET was combined with okra consumption, the results were similar, adding to a greater magnitude decrease of IL-6 in the liver, when compared to okra consumption alone. Thus, we conclude that okra consumption combined or not with aerobic ET can have a protective effect on the cardiac tissue of male rats with MetS.

### Study limitations

Our study is the first to present data on the effect of okra consumption on cardiac tissue and hemodynamic parameters, so we did not have the opportunity to compare the response with other studies. In addition, we used an animal model that has leptin resistance, which does not reflect the main mechanism of obesity in humans.

## Data Availability

The datasets used and/or analysed during the current study available from the corresponding author on reasonable request.
